# Computer-Based Assessment: Dual-Task Outperforms Large-Screen Cancellation Task in Detecting Contralesional Omissions

**DOI:** 10.3389/fpsyg.2021.790438

**Published:** 2022-01-07

**Authors:** Sanna Villarreal, Matti Linnavuo, Raimo Sepponen, Outi Vuori, Mario Bonato, Hanna Jokinen, Marja Hietanen

**Affiliations:** ^1^Division of Neuropsychology, HUH Neurocenter, Helsinki University Hospital and University of Helsinki, Helsinki, Finland; ^2^Department of Psychology and Logopedics, Faculty of Medicine, University of Helsinki, Helsinki, Finland; ^3^Department of Electrical Engineering and Automation, Aalto University, Espoo, Finland; ^4^Department of General Psychology, University of Padova, Padua, Italy

**Keywords:** neuropsychological evaluation, computer-based methods, paper-and-pencil tasks, dual-task, hemispatial neglect, stroke, neuropsychology, extinction

## Abstract

**Objective:** Traditionally, asymmetric spatial processing (i.e., hemispatial neglect) has been assessed with paper-and-pencil tasks, but growing evidence indicates that computer-based methods are a more sensitive assessment modality. It is not known, however, whether simply converting well-established paper-and-pencil methods into a digital format is the best option. The aim of the present study was to compare sensitivity in detecting contralesional omissions of two different computer-based methods: a “digitally converted” cancellation task was compared with a computer-based Visual and Auditory dual-tasking approach, which has already proved to be very sensitive.

**Methods:** Participants included 40 patients with chronic unilateral stroke in either the right hemisphere (RH patients, *N* = 20) or the left hemisphere (LH patients, *N* = 20) and 20 age-matched healthy controls. The cancellation task was implemented on a very large format (173 cm × 277 cm) or in a smaller (A4) paper-and-pencil version. The computer-based dual-tasks were implemented on a 15′′ monitor and required the detection of unilateral and bilateral briefly presented lateralized targets.

**Results:** Neither version of the cancellation task was able to show spatial bias in RH patients. In contrast, in the Visual dual-task RH patients missed significantly more left-sided targets than controls in both unilateral and bilateral trials. They also missed significantly more left-sided than right-sided targets only in the bilateral trials of the Auditory dual-task.

**Conclusion:** The dual-task setting outperforms the cancellation task approach even when the latter is implemented on a (large) screen. Attentionally demanding methods are useful for revealing mild forms of contralesional visuospatial deficits.

## Introduction

Hemispatial neglect is a heterogenous and multicomponential syndrome encompassing several attentional and spatial deficits (Husain, 2019). Neglect is often described as a directional bias in orienting attention. It is often associated to contralesional omissions occurring in contexts of competition for selection (ipsilesional stimuli are prioritized over contralesional ones; cf., extinction), as well as to non-lateralized attention deficits (Kerkhoff, 2001; Husain, 2019). Conventionally, contralesional spatial bias in visual attention has been assessed with paper-and-pencil methods (Kortte and Hillis, 2009). In particular, cancellation tasks are widely used and considered to be very sensitive (Halligan et al., 1991; Bowen et al., 1999; Ferber and Karnath, 2001; Azouvi et al., 2002; Parton et al., 2004). Patients with severe neglect typically fail to cross targets in the contralesional hemispace, even in the easiest versions of these tasks (Albert, 1973; Halligan et al., 1991). Patients with milder forms of the deficit, however, tend not to show omissions in cancellation tasks without distractors. To overcome this problem, task sensitivity has been enhanced by specifically adjusting the stimuli, for example, by increasing the target density and the relative salience of distractors (Rapcsak et al., 1989; Aglioti et al., 1997; Chatterjee et al., 1999; Sarri et al., 2009; Bickerton et al., 2011; Basagni et al., 2017; Ten Brink et al., 2020; Villarreal et al., 2020). To prevent compensation and to identify milder deficits, time limits are sometimes introduced (Priftis et al., 2019). Another possibility is to calculate indices that are more sophisticated than the simple number of omissions, such as the starting point analysis (Azouvi et al., 2002; Nurmi et al., 2010, 2018), which allows quantifying neglect patients’ tendency to start visual searching from the ipsilesional hemispace (Kinsbourne, 1987; Karnath, 1988; Olk et al., 2002).

Despite these efforts, several studies have demonstrated that computer tasks, which are complex, and require high attentional demands, are more sensitive than paper-and-pencil cancellation tasks (Deouell et al., 2005; List et al., 2008; Rengachary et al., 2009; Bonato et al., 2010, 2012; Blini et al., 2016; Andres et al., 2019). Chronic stroke patients who present perfectly normal performance even in the most difficult cancellation tasks show contralesional omissions when brief stimuli are presented concurrently with a secondary task (i.e., dual-task setting; Deouell et al., 2005; Bonato et al., 2010, 2012; Blini et al., 2016; Andres et al., 2019). Dividing attention and performing two simultaneous tasks necessitates executive control of attention (Cohen, 2014; Strobach et al., 2018). These aspects can sometimes be dramatically impaired in neglect (Mannan et al., 2005; Ronchi et al., 2009; Bonato et al., 2012). Neglect patients show perseveration errors (Mannan et al., 2005; Ronchi et al., 2009), and their executive dysfunction may also be seen as difficulty in allocating attentional resources (Cohen, 2014). This, in turn, may lead to less effective compensation in high demand dual-tasks, resulting in the emergence of otherwise “undetectable” deficits when the task is particularly demanding (Robertson and Frasca, 1992; van Kessel et al., 2013; Bonato, 2015; Strobach et al., 2018; Andres et al., 2019). This approach allows contrasting performance in a primary visuospatial task when performed alone or concurrently with another task (single vs. dual-task setting). It also allows presenting two concurrent targets (i.e., extinction setting) and overall requires higher attentional demands than cancellation tasks. However, computer-based applications have been criticized for introducing stimuli that subtend a limited visual angle. It has been claimed that tasks presented on a standard computer screen or on a sheet of paper may have poor ecological validity (Nakatani et al., 2013; Ulm et al., 2013). Advantages of assessment methods with large perceptual space have been described in studies using dual-tasks (van Kessel et al., 2013; Villarreal et al., 2020) and cancellation tasks (Tanaka et al., 2005; Tsirlin et al., 2009; Ulm et al., 2013; Knobel et al., 2020). Both the large-screen dual-tasks and the large-screen cancellation tasks appear to be more sensitive in revealing spatial bias than paper-and-pencil cancellation tasks (Ulm et al., 2013; van Kessel et al., 2013; Villarreal et al., 2020).

Past studies have highlighted the important role of unspecific attentional demands (Dawson et al., 2008; Bonato et al., 2010, 2012; Buxbaum et al., 2012; Blini et al., 2016; Ricci et al., 2016; Andres et al., 2019), large test fields (Nakatani et al., 2013; Ulm et al., 2013), or both (Villarreal et al., 2020) in determining the sensitivity of tests for neglect and extinction. In the present study, we aimed to capitalize on the previous findings by contrasting these two characteristics. We developed a new computer-based large-screen cancellation task and compared it with a computer-based dual-task approach with brief stimulus duration, which has already proven to be more sensitive than paper-and-pencil tasks in detecting contralesional omissions (Bonato et al., 2010, 2012; Bonato, 2015; Blini et al., 2016; Andres et al., 2019).

We aimed to examine whether contralesional omissions could be triggered more easily by using a cancellation task presented on a large perceptual space or by using attentionally demanding dual-tasks presented on a standard computer screen. These findings were also compared with those obtained with a paper-and-pencil version of the new cancellation task. Our hypothesis was that both the large-screen cancellation task and the dual-tasks are more sensitive than the paper-and-pencil cancellation task in revealing contralesional deficits in spatial processing.

## Materials and Methods

### Participants

The participants’ characteristics have been described in detail in our previous study (Villarreal et al., 2020). In short, 40 patients with a first-ever stroke diagnosis (ischemic, hemorrhagic, or both) from the Neurology Outpatient Clinic of the Helsinki University Hospital (HUH) and 20 healthy volunteers participated in the study during 2016–2019. Each patient’s stroke was neuroradiologically verified (CT/MRI). The scans were evaluated by an independent neuroradiologist, details of the lesion locations are presented in Supplementary Table A.

The patients were studied on average 106 days after hospital admission, and were recruited with the following exclusion criteria: previously diagnosed stroke, bilateral stroke, other neurological disease affecting cognition, visual field deficit according to clinical neurological or neuro-ophthalmological evaluation, primary impairment in hearing or sight (with two exceptions: hyperopia or myopia corrected with glasses), substance abuse, severe psychiatric disease, or severe motor or cognitive symptoms preventing participation. These exclusions were performed by consulting chief neuropsychologist (MH) for potentially eligible consecutive patients (*n* = 58) and then, with these patients’ written informed consent, based on the records of medical history accessed at HUH. The participants comprised three groups: the RH patient group (20 right hemisphere stroke patients, mean age 53 SD ± 8 yrs., 11 females), the LH patient group (20 left hemisphere stroke patients, mean age 51 SD ± 9 yrs., 5 females), and the Control group (20 healthy controls, mean age 46 SD ± 15 yrs., 12 females). The three groups did not differ statistically for age, gender, education, nor for self-reported symptoms of depression (Salokangas et al., 1995; see Villarreal et al., 2020). There were no statistically significant differences between the patient groups (RH vs. LH) in the number of days post-onset of stroke prior to the study or in stroke type (Villarreal et al., 2020). Demographic characteristics of the participants are presented in Table 1.

**TABLE 1:** Demographic and clinical characteristics of the participants (see also Villarreal et al., 2020).

Characteristics of the participants	LH patients	RH patients	Controls
Age; years^a^	51 (SD 9)	53 (SD 8)	46 (SD 15)
Gender; female / male^b^	25% / 75%	55% / 45%	60% / 40%
Handedness; left / right^b^	0% / 100%	5% / 95%	0% / 100%
Education; years^a^	16 (SD 4)	15 (SD 3)	16 (SD 3)
Depression scale score^a,1^	5 (SD 4)	5 (SD 4)	3 (SD 4)
Lesion type; haemorrhage / ischaemia / both^b^	5% / 90% / 5%	15% / 60 % / 25%	
Days post-onset of stroke prior to study^a^	105 (SD 42)	106 (SD 45)	
Rehabilitation sessions prior to study^a,2^	3 (SD 2)	3 (SD 2)	
Type of outpatient rehabilitation^b,3^	50% / 50%	70% / 30%	
Neglect diagnosed initially^b,4^	15%	55%	

*^a^Mean (standard deviation); ^b^Percentage.*

*^1^Scale consists of 10 items, score range 0 – 30 (Salokangas et al., 1995).*

*^2^Neuropsychological outpatient rehabilitation.*

*^3^Multidisciplinary rehabilitation / only neuropsychological rehabilitation.*

*^4^Based on clinical neuropsychological assessment conducted on average 13 (SD 15) days post stroke.*

*LH, left hemisphere stroke; RH, right hemisphere stroke; and SD, standard deviation.*

Initial assessment of the patients’ visual neglect was based on clinical neuropsychological examination, which was conducted during acute ward care, on average 13 days (SD 15) post stroke. At this early stage of the recovery, 55% of the RH patients and 15% of the LH patients were diagnosed with neglect. Details of the initial assessment are presented in Supplementary Table A. At the time of the study, the patients’ neglect was assessed with the traditional cancellation task, the Bells Test (Gauthier et al., 1989; see Villarreal et al., 2020). Based on traditional criteria (i.e., six or more contralesional omissions in the Bells Test; Gauthier et al., 1989), none of the RH or LH patients showed visual neglect (see Supplementary Table A). LH and RH patients’ performance was also comparable in other neuropsychological test variables. However, the patient groups differed statistically significantly from controls in several tests assessing linguistic and executive functions, and processing speed (see Villarreal et al., 2020). To specify, controls were faster than RH patients in the Trail Making Test A. They also performed better than RH patients in semantic fluency, and better than both patient groups in design fluency. LH patients were worse than controls in phonemic fluency, and in the Bourdon–Wiersma dot cancellation single task, as well as in the dot cancellation and in the number count dual-tasks.

All patients received neuropsychological outpatient rehabilitation at the time of their participation. Details of the rehabilitation are presented in Table 1.

### Procedure

Each participant performed three computer-based tasks and a paper-and-pencil version of the new cancellation task. The order of the computer tasks was fixed: (1) the large-screen cancellation task, (2) the Visual dual-task, and (3) the Auditory dual-task. Patients performed paper-and-pencil cancellation tasks in multiple sessions during rehabilitation. The control participants performed all tasks in the same session.

**FIGURE 1:**
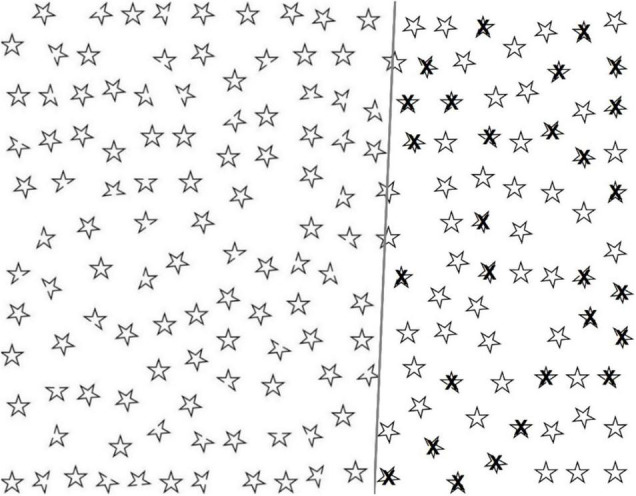
The large-screen Twinkle Task: demonstrations of empty task sheet (left), and correctly selected targets (right).

As previously described (Villarreal et al., 2020, 2021), LH patients in our sample showed no spatial bias in visual attention, and RH patients showed only subtle neglect, which was not evident in the traditional cancellation task, the Bells Test. Therefore, in the present study, we used more sophisticated approaches for data analysis to potentially increase the sensitivity of the new cancellation task. We analyzed the mean position of hits (Toraldo et al., 2017) and the starting points and recorded the performance times (see the section “Data Analyses”). To improve task sensitivity, we increased visuoperceptual demands by using high density targets, distractors that were similar to the target (see Figure 1), and limited time for visual searching in the large-screen version.

#### Cancellation Tasks

A new cancellation task, the Twinkle Task, was developed, and described here for the first time, by combining the original ideas of Wilson et al. (1987) with those of Ota et al. (2001). Wilson et al. (1987) introduced a star cancellation task to assess visual neglect; it contains letters, words, and five-pointed stars in two different sizes arranged randomly on an A4 size paper. Patients are asked to cancel all the small stars. Ota et al. (2001) presented a cancellation task to differentiate body-centered and stimulus-centered neglect. The task contains circles and pseudo-circles arranged in a random manner on an A4 size paper. There is a missing portion (“the gap”) in the pseudo-circles on either the right or left side. The task is to circle every complete circle and to cross out every incomplete pseudo-circle. Subsequently, other “gap detection tasks” have also been introduced (see, e.g., Parton et al., 2006; Chechlacz et al., 2010; Bickerton et al., 2011; Demeyere et al., 2015; Mancuso et al., 2015).

The Twinkle Task comprises 180 five-pointed star-shaped black outline figures arranged in a pseudo-random array on a white background (see Figure 1). There are 110 intact figures (distractors) and 70 figures with one point missing (targets, open cut end, “the gap”). All figures are of equal size. The task is to select the targets. Equal numbers (31) of targets are located on the left and right sides (covering 46 and 46% of the total area), and 8 in the central area (covering 8% of the total area). Thirty (43%) targets have a missing point on the left side, 30 (43%) on the right side, and 10 (14%) in straight-up or straight-down positions.

##### Large-Screen Version of the Cancellation Task

Active Space, a relatively recent application, was used to generate a large-screen version of the Twinkle Task (Linnavuo et al., 2010; Rimminen et al., 2010). Detailed technical properties of the Active Space have been described in previous studies (Linnavuo et al., 2010; Rimminen et al., 2010; Villarreal et al., 2020). In brief, the visual stimuli were generated by a short throw video projector (Epson EB-680, Seiko EPSON Corporation, Suwa, Japan) producing a wall-sized screen (173 cm × 277 cm) with the midpoint located 120 cm from the floor. The pixel size was 1.9 mm × 1.9 mm. Participants sat in front of the screen at 180 cm distance. The visual angle covered was approximately 51 degrees vertically and 75 degrees horizontally. The control of the application and the task were implemented using LabVIEW systems engineering software (National Instruments, Austin, TX, United States).

Participants performed the task by using a mouse with their dominant hand to move an on-screen cursor. Targets had to be selected by pressing the left button of the mouse. The maximum width of each star figure was 10 cm (about 1 degree). The selected target was marked with a black cross on the screen, and the selection could not be canceled. The participants were not informed about the number of targets. They were instructed to perform the task as quickly and precisely as possible and to inform the experimenter as soon as they believed that all targets were selected. The experimenter then immediately terminated the task, which would otherwise disappear automatically after a 3-min time limit (patients were informed about this time constraint). Correct and false selections, omissions, and performance time were registered by the software. To ensure that participants understood the task instructions, a 30-s practice trial with verbal guidance and feedback preceded the actual test session. In the practice trial, there were 6 targets displayed in the central area of the screen.

##### Starting Points in the Large-Screen Cancellation Task

**FIGURE 2:**
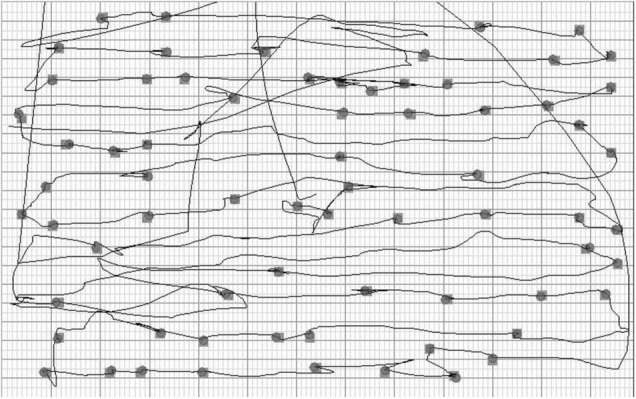
The large-screen Twinkle Task: visualization of the scanning pattern and the selected targets in a representative patient.

The individual scanning pattern and the x and y coordinates of the selected targets were registered on the computer (Figure 2). The display size was 1,280 horizontal (*x* coordinate from left to right) and 1,024 vertical (*y* coordinate from top to bottom) pixels. The starting point of each participant was coded according to the horizontal location of the first selected target. The targets were evenly distributed so that the *x* coordinate of the left-most target was 38 pixels and the right-most 1,254 pixels.

##### Paper-and-Pencil Version of the Cancellation Task

The paper-and-pencil version of the Twinkle Task consisted of a screenshot of the large-screen version, with identical stimuli and proportions, presented on an A4 size paper. The maximum width of each star was 0.8 cm. The participant was seated at a desk. The task sheet was placed on the desk and aligned with the midsagittal plane of the participant’s body. Each participant was instructed to mark all target stars as quickly and precisely as possible with a pen held in their dominant hand and to inform the experimenter as soon as they believed that all targets were found. The experimenter then immediately terminated the task and manually stopped a timer. Performance time was not limited.

#### Computer-Based Dual-Tasks

**FIGURE 3:**
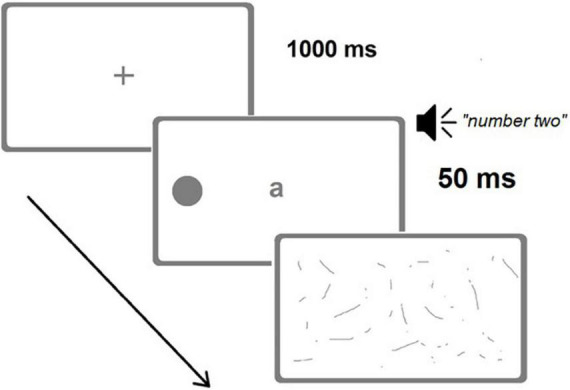
A representative trial of the Visual dual-task (image not in scale). A left target (alternative positions possible: right or bilateral) is briefly presented (50 ms) concurrently with a central letter and an auditory digit. Correct responses for the Visual dual-task would have been “a” (central letter) and “left” (target position), while for the Auditory dual-task they would have been “four, six” (count in steps of two from the auditorily presented “two”) and “left.” (Adapted from Bonato, 2015).

Participants performed a Finnish version of the two computer-based dual-tasks developed by Bonato et al. (2010). E-Prime (Psychology Software Tools, Sharpsburg, PA, United States^1^) was utilized in programming and administrating the tasks. The method has previously been described in detail (Bonato et al., 2010, 2012; Bonato, 2015). In a nutshell, it allows measuring performance accuracy in detecting briefly presented, lateralized targets while concurrently processing another (visual or auditory) stimulus (see Figure 3 for a representative trial).

Participants sat at a distance of 60 cm from a 15′′ screen. Each trial started with a blank display (1,000 ms) followed by a black central fixation cross (1,000 ms). Then, three different stimuli were presented concurrently: black dot target(s) (diameter 8 mm, flash time 50 ms), a central letter (font size 38, same duration, *a*, *b*, *v*, or *z*; one in each trial), and a spoken number (*one*, *two*, *eight*, or *nine* in Finnish; one number in each trial). The dot target(s) was located unilaterally (135 mm, 12.8° to the left or right from the central fixation point) or bilaterally (at the left and right simultaneously). One target type (left unilateral, right unilateral, or bilateral) appeared in each trial, and there were 48 trials altogether (16 of each target type presented in a pseudorandom manner).

In the Visual dual-task, the participant was instructed to read the central letter aloud and then to verbally report the location of the dot target(s), ignoring the spoken number. In the Auditory dual-task, the participant was instructed to start with the spoken number (e.g., 9) by counting forward twice in steps of two (e.g., 9, 11, 13), and then verbally report the position of the dot target(s) disregarding the central letter. Verbal guidance and a training session preceded the actual tests. The experimenter entered the participant’s verbal responses (the location of the dot target(s) as being “left,” “right,” “both sides,” or “no response”). Response time was not restricted. A mask-like black-and-white screen followed the flash of the visual stimuli and continued through the spoken number presentation until the participant’s response was coded.

A unilateral target dot was interpreted as “omitted” if the participant did not respond or if they provided a wrong response to the target location. With bilateral targets, “left omission” meant that the participant had incorrectly reported a target location as being “on the right,” while actually appearing bilaterally (cf. left-sided extinction); correspondingly, “right omission” meant that the target was reported as being located “on the left,” while actually appearing bilaterally (cf. right-sided extinction).

### Data Analyses

Variables of the large-screen and paper-and-pencil Twinkle Tasks were created by calculating each participant’s left-sided and right-sided *omissions*. The spatial distribution of correctly canceled targets (hits) was determined by extracting *the mean position of hits* (MPH). MPH, developed by Toraldo et al. (2017), is a standardized statistical procedure to statistically test whether there is a spatial imbalance in cancellation performance. In the present study, the value of the MPH was determined from the electronic image of the Twinkle Task sheet. MPH was the standardized value of the mean horizontal location (*x* coordinate in pixels) of the selected targets. The range of the MPH varied from −0.5 (the extreme left) to +0.5 (the extreme right). Zero indicated that correctly detected targets were distributed equally between the left and right hemifields. Positive values indicated rightward spatial bias (i.e., predominance of left-sided omissions), and correspondingly, negative values indicated leftward spatial bias (i.e., predominance of right-sided omissions). *The starting point* variable of the large-screen Twinkle Task was created by determining the horizontal location (x coordinate in pixels) of the first target star marked by the participant. *The performance time* was determined for both the large-screen and the paper-and-pencil Twinkle Tasks. *Variables of the Visual and Auditory dual-tasks* were created by calculating each participant’s omissions separately for the unilateral and bilateral trials and for the left and right hemifields.

The Statistical Package for Social Sciences (IBM SPSS Statistics for Windows, Version 25.0, Armonk, NY, United States: IBM Corporation) was used for statistical analyses. Nonparametric methods were used because of skewed distributions. Between-groups comparisons were analyzed with the Kruskal–Wallis test (χ^2^). Dunn’s test was used for *post-hoc* analyses. For multiple pairwise comparisons, *p*-values were adjusted using Bonferroni correction. Within-group analyses (omission differences between the two hemifields and performance time differences between the two versions of the Twinkle Task) were performed using the Wilcoxon signed-rank test. Effect sizes were calculated by computing eta squared (η^2^) for the Kruskal–Wallis test and *r* for the Wilcoxon signed-rank test (Tomczak and Tomczak, 2014). For statistically significant group differences, Cohen’s descriptions for η^2^ (large effect: 0.14, medium effect: 0.06, small effect: 0.01) and for *r* (large effect: 0.5, medium effect: 0.3, small effect: 0.1) were used (Cohen, 1988). The level of statistical significance was set at 0.05.

## Results

### Large-Screen (and Paper-and-Pencil) Cancellation Tasks

#### Omissions

Average omissions and related statistical analyses for the two versions of the Twinkle Task are presented in Table 2, separately for each group. In the large-screen version, there were no statistically significant differences between the groups in left-sided omissions. In contrast, LH patients made statistically significantly more right-sided omissions than controls (2% vs. 0%). No statistically significant differences were observed for right-sided omissions between the RH patients and controls, nor between the patient groups. In the paper-and-pencil version, there were no statistically significant differences between the groups in left-sided nor in right-sided omissions.

**TABLE 2:** Average omissions, and related statistical group comparisons in the two versions of the cancellation task (computerized with large screen or paper and pencil).

The twinkle task								
Omission comparisons between the participant groups^a^		LH	RH	C	Statistics	df	*p* value^b^	Effect size^c^
		Average omissions (%)						
Large-screen version, left hemifield		1 %	1 %	1 %	0.670	2	0.715	
Large-screen version, right hemifield		2 %	2 %	0 %	6.190	2	0.045	η^2^ = 0.074**
Mean ranks		35.62	31.02	24.85				
*Post hoc* comparisons	RH vs. C				−6.175		0.466	
	RH vs. LH				4.600		0.870	
	C vs. LH				−10.775		0.040	*r* = 0.392**
Paper-and-pencil version, left hemifield		2%	2%	1%	1.714	2	0.424	
Paper-and-pencil version, right hemifield		1%	1%	1%	1.108	2	0.575	
Omission comparisons between the two hemifields^d^								
Large-screen version	LH				−1.732		0.083	
	RH				−0.108		0.914	
	C				−0.707		0.480	
Paper-and-pencil version	LH				−0.209		0.834	
	RH				−1.155		0.248	
	C				−1.134		0.257	

*^a^Kruskal–Wallis test (χ^2^), mean ranks, post hoc comparisons, and effect sizes presented for significant group-differences.*

*^b^For multiple pairwise comparisons, p values adjusted by the Bonferroni correction.*

*^c^Effect sizes according to Cohen (1988).*

η^2^ = *small >0.01, **medium >0.06, ***large >0.14 and r = *small >0.1, **medium >0.3, ***large >0.5.

*^d^Wilcoxon signed-rank test (Z).*

*LH, left hemisphere stroke patients; RH, right hemisphere stroke patients; and C, control participants.*

Omission comparisons between the left and right hemifields within the groups revealed no statistically significant differences in the LH or RH patients or the controls in either version of the Twinkle Task.

#### Mean Position of Hits

The average mean position of hits (MPHs) in the two versions of the Twinkle Task and related statistical analyses are presented in Table 3. There were no statistically significant differences between the groups in the MPHs of the large-screen or the paper-and-pencil task. That is, neither conventional nor computerized versions detected any pattern of contralesional omissions in the sample.

**TABLE 3:** Average mean position of hits (MPHs) and starting points in the cancellation tasks, and related statistical group comparisons.

The twinkle task						
Average MPHs and statistical group comparisons^a^	LH	RH	C	Statistics^e^	df	*p* value
Large-screen version: average horizontal MPHs^b^	−0.00036	−0.00067	−0.00021	1.572	2	0.456
Paper-and-pencil version: average horizontal MPHs^b^	0.00144	0.00096	0.00110	0.148	2	0.929

Average starting points and statistical group comparisons^c^						
Large-screen version: average horizontal starting points^d^	230	318	260	3.823	2	0.148

*^a^MPH, mean position of hits; i.e. the standardized value of the mean horizontal location (x coordinate in pixels) of the selected targets across the task sheet.*

*^b^The range of the MPH varies from −0.5 (the extreme left) to +0.5 (the extreme right).*

*^c^Starting point is coded according to horizontal location (x coordinate in pixels) of the first selected target.*

*^d^The range of the starting point varies from 38 (the extreme left) to 1,254 (the extreme right) pixels.*

*^e^Kruskal–Wallis test (χ^2^).*

*LH, left hemisphere stroke patients; RH, right hemisphere stroke patients; and C, control participants.*

#### Starting Points

Average horizontal starting points in the large-screen version of the Twinkle Task and related group-comparisons are presented in Table 3. There were no statistically significant differences between the participant groups in horizontal starting points.

#### Performance Times

Average performance times of the two versions of the Twinkle Task and related statistical analyses are presented in Table 4. There were no statistically significant differences between the groups in performance times of the large-screen version. However, in the paper-and-pencil version, RH patients were significantly slower than controls (135 s vs. 95 s). LH patients did not differ statistically significantly from controls, and the two patient groups did not differ statistically from each other in performance times on the paper-and-pencil version.

**TABLE 4:** Average performance times in the two versions of the cancellation task, and group comparisons.

The twinkle task								
Performance time comparisons between the participant groups^a^								
		LH	RH	C	Statistics	df	*p* value^b^	Effect size^d^
		Average performance time, s (SDs)						
Large-screen version (time limit of 180 s)		137 (32)	146 (29)	127 (28)	3.481	2	0.175	
Paper-and-pencil version (no time limit)		113 (29)	135 (51)	95 (21)	10.206	2	0.006	η^2^ = 144***
Mean ranks		31.68	38.03	20.70				
*Post hoc* comparisons	C vs. RH				17.326		0.005	*r* = 0.498**
	LH vs. RH				6.351		0.745	
	C vs. LH				10.975		0.130	

Performance time comparisons between the two versions of the cancellation task^c^								
	LH				−2.688		0.007	*r* = −0.601***
	RH				−1.248		0.212	
	C				−3.397		0.001	*r* = −0.760***

*^a^Kruskal-Wallis test (χ^2^), mean ranks, post hoc comparisons, and effect sizes presented for significant group-differences.*

*^b^For multiple pairwise comparisons, p values adjusted by the Bonferroni correction.*

*^c^Wilcoxon signed-rank test (Z).*

*^d^Effect sizes according to Cohen (1988).*

η^2^ = *small >0.01, **medium >0.06, ***large >0.14 and r = *small >0.1, **medium >0.3, ***large >0.5.

*Data for 1 patient missing; LH, left hemisphere stroke patients; and RH, right hemisphere stroke patients.*

*C, control participants, SD, standard deviation.*

The large-screen version took longer to be completed than the paper-and-pencil version for LH patients (137 s vs. 113 s) and for controls (127 s vs. 95 s). In contrast, RH patients’ performance times did not differ statistically significantly between the two task versions. Two RH patients, one LH patient, and one control participant failed to perform the large-screen version within the 3-min time limit.

### Computer-Based Visual and Auditory Dual-Tasks

#### Visual Dual-Task

**FIGURE 4:**
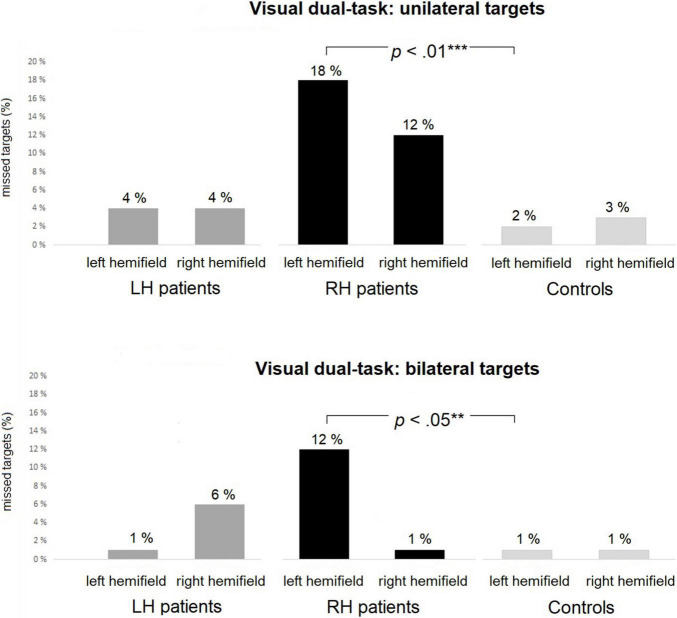
The Visual dual-task: average percentages of missed targets within each group in unilateral **(top panel)** and bilateral **(bottom panel)** trials. RH patients missed significantly more left-sided targets than controls in both the unilateral and bilateral trials. Asterisks represent effect size (***large effect; **medium effect).

Average omissions and related statistical group comparisons for the Visual dual-task are presented in Table 5. RH patients missed significantly more left-sided targets than did controls in both the unilateral and bilateral trials (18% vs. 2% and 12% vs. 1%, respectively, see Figure 4). No statistically significant differences were observed in right-sided omissions between the RH patients and controls. There were no statistically significant differences in left-sided or right-sided omissions between LH patients and controls, nor between the patient groups.

**TABLE 5:** Average omissions in the visual dual-tasks, and related group comparisons, shown separately for unilateral/bilateral trials and for each hemifield.

Visual dual-task								
Average omissions (%) and related group-comparisons^a^								
		LH	RH	C	Statistics (χ^2^)	df	*p* value^b^	Effect size^c^
Unilateral targets								
Left hemifield		4%	18%	2%	10.729	2	0.005	η^2^ = 0.153***
Mean ranks		30.73	38.65	22.12				
*Post hoc* comparisons	C vs. RH				16.525		0.003	*r* = 0.518***
	LH vs. RH				7.925		0.349	
	C vs. LH				8.600		0.265	
Right hemifield		4%	12%	3%	3.715	2	0.156	
Bilateral targets								
Left hemifield		1%	12%	1%	8.253	2	0.016	η^2^ = 0.110**
Mean ranks		28.75	36.70	26.05				
*Post hoc* comparisons	C vs. RH				10.650		0.017	*r* = 0.437**
	LH vs. RH				7.950		0.117	
	C vs. LH				2.700		1.000	
Right hemifield		6%	1%	1%	3.296	2	0.192	

*^a^Kruskal–Wallis test (χ^2^), mean ranks, post hoc comparisons, and effect sizes presented for significant group-differences.*

*^b^For multiple pairwise comparisons, p values adjusted by the Bonferroni correction.*

*^c^Effect sizes according to Cohen (1988).*

η^2^ = *small >0.01, **medium >0.06, ***large >0.14 and r = *small >0.1, **medium >0.3, ***large >0.5.

*LH, left hemisphere stroke patients; RH, right hemisphere stroke patients; and C, control participants.*

Statistical comparisons between left-sided and right-sided omissions within the groups are presented in Table 6. There were no statistically significant differences between left-sided and right-sided omissions within any of the groups.

**TABLE 6:** Comparisons between left-sided and right-sided omissions in Visual and Auditory dual-tasks within each group.

Visual and auditory dual-tasks							
R and L omission comparisons^a^							
		Unilateral targets			Bilateral targets		
		Statistics (Z)	*p* value		Statistics (Z)	*p* value	Effect size (*r*)^b^
Visual dual-task	LH	−1.207	0.227	LH	−1.403	0.161	
	RH	−1.712	0.087	RH	−1.921	0.055	
	C	−1.265	0.206	C	−0.557	0.577	
Auditory dual-task	LH	−0.586	0.558	LH	0.000	1.000	
	RH	−0.359	0.719	RH	−2.503	0.012	−0.56***
	C	−0.587	0.557	C	−1.134	0.257	

*^a^Wilcoxon signed-rank test, effect sizes presented for significant differences.*

^b^Effect sizes according to Cohen, 1988: r = *small >0.1, **medium >0.3, ***large >0.5.

*R, right hemifield, L, left hemifield, and LH, left hemisphere stroke patients.*

*RH, right hemisphere stroke patients, and C, control participants.*

#### Auditory Dual-Task

Average omissions and related group comparisons for the Auditory dual-task are presented in Table 7. The groups did not differ statistically significantly in the left-sided or right-sided omissions for unilateral trials. Omission comparisons of the bilateral trials indicated a statistically reliable group difference in left-sided omissions (2% vs. 7% vs. 1% for LH and RH patients, and controls, respectively), but the finding was not backed up by pairwise comparisons. There were no statistically significant group differences in right-sided omissions for bilateral trials.

**TABLE 7:** Average omissions in the Auditory dual-tasks, and related group comparisons, shown separately for unilateral/bilateral trials and for each hemifield.

Auditory dual-task								
Average omissions (%) and related group-comparisons^a^								
		LH	RH	C	Statistics (χ^2^)	df	*p* value^b^	Effect size^c^
Unilateral targets								
Left hemifield		3%	6%	2%	4.040	2	0.133	
Right hemifield		5%	6%	2%	1.617	2	0.445	
Bilateral targets								
Left hemifield		2%	7%	1%	6.033	2	0.049	η^2^ = 0.071**
	mean ranks	27.68	36.30	27.52				
*Post hoc* comparisons	C vs. RH				8.775		0.096	
	LH vs. RH				8.625		0.105	
	C vs. LH				0.150		1.000	
Right hemifield		4 %	1 %	0 %	0.477	2	0.788	

*^a^Kruskal–Wallis test (χ^2^), mean ranks, post hoc comparisons, and effect sizes presented for significant group-differences.*

*^b^For multiple pairwise comparisons, p values adjusted by the Bonferroni correction.*

^c^Effect sizes according to Cohen (1988): η^2^ = *small >0.01, **medium >0.06, ***large >0.14.

*LH, left hemisphere stroke patients; RH, right hemisphere stroke patients; and C, control participants.*

**FIGURE 5:**
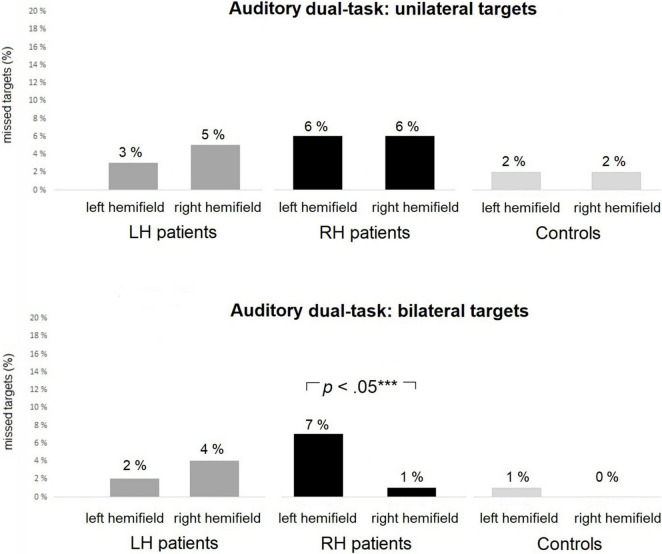
The Auditory dual-task: average percentages of missed targets within each group in unilateral **(top panel)** and bilateral **(bottom panel)** trials. RH patients missed significantly more left-sided than right-sided targets in bilateral trials. Asterisks represent effect size (***large effect).

Statistical analyses between left-sided and right-sided omissions within the groups are presented in Table 6. There were no statistically significant differences between the left-sided and right-sided omissions for unilateral trials within any of the groups. However, in bilateral trials, RH patients missed significantly more left-sided than right-sided targets (7% vs. 1%, see Figure 5). There were no statistically significant differences between the left-sided and right-sided omissions for bilateral trials in LH patients or controls.

## Discussion

The aim of the present study was to examine whether visuospatial attention deficits could be identified more sensitively by using the digital and enhanced versions of a cancellation task or by using attentionally demanding computer-based tasks requiring participants to divide their attention. To answer the study question, we developed a novel large-screen cancellation task, the Twinkle Task, and compared it with a more established dual-task approach with briefly presented lateralized targets. The findings of these computer methods were also compared to those obtained with a traditional paper-and-pencil version of the Twinkle Task. We predicted that both the large-screen and the dual-task computer methods should have been more sensitive than paper-and-pencil tasks in triggering contralesional omissions. Performance was compared across two groups of stroke patients (left hemisphere LH or right hemisphere RH) and one group of healthy, matched controls.

Our main finding is that RH patients showed no spatial bias in either version of the Twinkle Task but showed contralesional omissions in the dual-task regardless of whether it was visual or auditory in nature. We then reasoned that maybe more sensitive measures (i.e., the starting points and the mean position of hits of the Twinkle Task) might have revealed spatial bias also in cancellation tasks. Additional analyses on these measures did not reveal any spatial bias in cancellation tasks. The computer-based dual-task allowed to draw a different picture. RH patients missed significantly more left-sided targets than controls in both the unilateral and bilateral trials of the Visual dual-task. They also missed significantly more left-sided than right-sided targets in the Auditory dual-task but only for bilateral trials. LH patients missed more right-sided targets than controls in the large-screen Twinkle Task, but not in the paper-and-pencil version nor in the dual-tasks. Both LH patients and controls were slower in the large-screen version compared to the paper-and-pencil version of the Twinkle task. This phenomenon did not become evident in RH patients who were already slower than controls in the paper-and-pencil version.

The findings of the present study expand on those of previous studies (Bonato et al., 2010, 2012; Bonato, 2015; Andres et al., 2019; Villarreal et al., 2020), indicating that coupling a dual-task setting with brief stimulus duration is a sensitive approach, which triggers contralesional omissions in patients with chronic right hemisphere stroke. This might be since using compensatory strategies to cope with spatial attention deficits is challenging in those situations characterized by the need to simultaneously process of two/three distinct aspects, and the time allowed for target detection is limited (Russell et al., 2004; Bonato and Deouell, 2013; Andres et al., 2019). Therefore, as stated in previous studies, unspecific attentional demands appear to be among the most important factors in increasing assessment sensitivity (Robertson and Frasca, 1992; Schendel and Robertson, 2002; Bonato, 2012, 2015; Ricci et al., 2016). If attentional demands are high enough, a task presented on a standard computer screen allows identifying even subtle deficits in contralesional spatial processing. This finding is important and might positively impact clinical practice, as in hospitals and rehabilitation centers ordinary hardware for assessment purposes is more easily available than additional technology. Nevertheless, a large test field, if available, presumably increases ecological validity (Nakatani et al., 2013), and along with a dual-task setting, it provably reveals even subtle neglect (Villarreal et al., 2020).

According to the present findings, cancellation tasks would not appear to be sufficiently demanding in detecting contralesional omissions in right hemisphere stroke. RH patients did not show spatial bias in either version of the Twinkle Task, even though the increase in the visual angle was massive and performance was analyzed with multiple parameters in addition to omissions. Unlike in previous studies (Azouvi et al., 2002; Nurmi et al., 2010, 2018; Basagni et al., 2017; Priftis et al., 2019), analyzing starting points, increasing perceptual demands, or limiting performance time had no sensitivity increment effect in detecting spatial bias in the group of RH patients. Standard, paper-and-pencil approaches are maximally informative in acute stroke, and with rather severe cases. However, our study confirms that in chronic right hemisphere stroke resulting in subtle symptoms, attentionally more demanding measures than cancellation tasks are needed (Taylor, 2003; Pedroli et al., 2015; Gammeri et al., 2020).

Post-stroke spontaneous recovery, rehabilitation, and learning effects might have contributed to modulating the performance of the patients. Fifty five percent of the RH patients were diagnosed with neglect initially (13 days post stroke) by using traditional methods. However, at the time of the study (106 days post stroke), none of the patients scored below the cut-off in the Bells Test. This might be considered compatible with the idea that spontaneous neurobiological recovery is maximal in the first 2–3 months post stroke (Nijboer et al., 2013). It warrants mentioning that patients in our sample additionally received rehabilitation and that they underwent assessments with cancellation tasks already in the early stages of recovery (see Supplementary Table A; Schendel and Robertson, 2002; Parton et al., 2004; Danckert and Ferber, 2006). Therefore, patients may quickly learn to compensate for mild deficits since the test type is familiar and static, and attentional demands are low (Erez et al., 2009; Hasegawa et al., 2011; Ogourtsova et al., 2017; Spreij et al., 2020).

A rather surprising yet logical finding was that LH patients missed slightly more right-sided targets than controls in the large-screen Twinkle Task. It is possible that the Twinkle Task is visuoperceptually more challenging than some classical cancellation tasks. The relative number of targets is greater, and they more closely resemble the distraction stimuli, for example, than in the Bells Test (Gauthier et al., 1989; see also Basagni et al., 2017). Some studies have reported that patients with left hemisphere stroke show reduced perceptual capacity (Woods et al., 2006). This may reflect an attentional deficit (Schendel et al., 2016) since perceptually demanding processing necessitates the engagement of attention (Aglioti et al., 1997). Possibly as a consequence, increments in visuoperceptual demands increase the sensitivity of neglect assessment (Riddoch and Humphreys, 1987; Aglioti et al., 1997; Basagni et al., 2017). Therefore, it could be hypothesized that the visuoperceptually demanding Twinkle Task brought up LH patients’ hidden neglect. This interpretation seems unlikely, however, since LH group showed no signs of neglect nor extinction in any of the computer-based dual-tasks in the present nor the previous study (Villarreal et al., 2020). These same dual-tasks, however, brought up subtle neglect or extinction in RH group. Besides, the present dual-tasks are also known to be sensitive in revealing subtle neglect and extinction in chronic left hemisphere stroke patients (Bonato et al., 2010; Blini et al., 2016). It should also be borne in mind that omission rate in the large screen Twinkle Task was identical for LH and RH group (2%), but only LH group’s performance differed statistically significantly from controls. These facts speak against our original hypothesis of the large-screen version being more sensitive than the paper-and-pencil version (even though the statistical finding in LH patients supports it). Therefore, the possibility of type 2 error cannot be excluded.

On the other hand, at the single case level, two of the LH patients showed statistically significantly more right-sided than left-sided omissions in the dual-tasks (see Supplementary Table A). These patients were not diagnosed with neglect initially by using traditional methods indicating that their contralesional attention deficits were subtle already in the early stage of recovery. It is also possible that these deficits were initially omitted e.g., due to prominent linguistic deficits (Kleinman et al., 2007). Left hemisphere stroke patients’ neglect is often underdiagnosed and, therefore, should be assessed with sensitive methods specifically designed for these patients (Kleinman et al., 2007; Blini et al., 2016).

Right hemisphere patients performed significantly slower than controls in the paper-and-pencil version of the Twinkle Task. LH patients and controls, on the other hand, were slower in the large-screen than the paper-and-pencil version, even though only the large-screen version had a time limit. It is possible that the large-screen version was more demanding than the paper-and-pencil version and, therefore, LH patients and controls took longer in completing the task. RH patients, on the other hand, performed at a consistently slower pace since no differences in performance times between the two task versions within the RH patients were found. This finding is supported by past studies, where patients with right hemisphere damage have shown slow processing speed, presumably as a remnant of neglect (Samuelsson et al., 1998; Bartolomeo and Chokron, 1999; Buxbaum et al., 2004; Viken et al., 2014; Nurmi et al., 2018).

Two limitations of the present study were that the patients showed only very subtle deficits and that the sample size was limited. As the RH patients’ visuospatial attention deficits were only subtle, they became more clearly evident in bilateral than unilateral trials, i.e., as an extinction. While the phenomenon was seen here in the dual-tasks, RH patients also showed extinction in the single task in our previous study (Villarreal et al., 2021). Our first study (Villarreal et al., 2020) demonstrated that the RH patients (but not LH patients) showed statistically reliable contralesional omissions for unilateral targets as well, in two different dual-tasks. This was present as prominent left-sided omissions when compared to those of the controls, but also in RH patients as statistically significantly more left-sided than right-sided omissions. These findings are in line with the view of extinction being one of the several sub-symptoms of neglect or co-occurring with the syndrome (Brozzoli et al., 2006; Cohen, 2014; Husain, 2019). However, there are also studies suggesting anatomo-functional dissociations between neglect and extinction (Neppi-Modona, 1999; Karnath and Rorden, 2012). As the dual-task method offers identical stimuli across different conditions, future studies with larger sample size might allow determining whether neglect and extinction are a continuum or whether they are independent. RH patients also showed ipsilesional omissions in the dual-tasks (although not statistically significant). The phenomenon may be explained by general inattention, which commonly co-occurs with neglect (Hjaltason et al., 1996; Husain and Rorden, 2003). In fact, our previous study (Villarreal et al., 2021) explicitly demonstrated that the RH patients were suffering from general inattention. LH group was suffering from general visual inattention as well but did not show neglect or extinction (Villarreal et al., 2020, 2021). Therefore, it is likely that RH group’s statistically reliable contralesional omissions are due to some specific consequence of right hemisphere damage. These findings may be explained by characteristics of the patients’ strokes (see Supplementary Table A), and are in line with the model of Husain (2019) suggesting that neglect is a multicomponential, rather heterogenous, syndrome. As the attention deficits were only subtle, and the sample size was limited, additional studies are needed to confirm the present results. Additional studies would also be needed to answer pertinent questions related to the new cancellation task: (1) was the Twinkle Task in itself sensitive and a useful screening task for clear neglect in the acute phase of stroke; (2) could it, much like some other “gap-detection” tasks (e.g., Ota et al., 2001), be useful in differentiating between body-centered and stimulus-centered neglect; and (3) could the scanning pattern recordings enabled by the large-screen version provide insights into the abnormal scanning latency and crossing index, which are typical for neglect patients (Rabuffetti et al., 2002; Ulm et al., 2013)?

Limitations of the present study also include the fact that we solely focused on deficient processing of peripersonal and extrapersonal space. Therefore, other subtypes (e.g., neglect in other sensory modalities, personal, motor, or premotor neglect) were not assessed. The presence/absence of these other subtypes might have influenced the findings. Even though the patients showed only subtle deficits in extrapersonal/peripersonal space, and many of them had recovered with respect to initial assessment, they still might have suffered from personal neglect. In the initial assessment, several RH patients showed indications of personal neglect (Supplementary Table A), and some evidence suggest that recovery for this type of neglect can be slower than, and independent of, extrapersonal neglect. In the study of Iosa et al. (2016), recovery rate after a physiotherapeutic and a six-week neuropsychological rehabilitation was nearly 80% for extrapersonal neglect but only 58% for mild personal neglect. Recoveries from these neglect subtypes did not correlate. Finally, as the present patients were not assessed for motor or premotor neglect, or mild physical deficits, their possible effect on the cancellation task performance remains elusive given that the responses imply directional movements (Na et al., 1998). However, as the omission rate in the cancellation tasks was low, and slow performance was seen only in RH patients, whose dominant hand was largely unaffected by stroke (see Table 1), this limitation seems to be uninfluential in practical terms.

We only used the Bells cancellation task as a traditional test to assess the patients’ visuospatial attention deficits (see Villarreal et al., 2020) while often a battery of tests is used [e.g., Behavioral Inattention Test (BIT) by Wilson et al. (1987)]. Therefore, the conclusion of the computer dual-tasks being more sensitive than traditional tests is limited to cancellation tasks only. However, several previous studies (Bonato et al., 2010, Bonato et al., 2013; Bonato, 2015; Blini et al., 2016) have demonstrated with chronic unilateral stroke patients that the computer dual-tasks used in the present study are more sensitive in identifying subtle neglect than various traditional tests (e.g., conventional BIT, and even specific versions of paper-and-pencil cancellation tasks adapted to make them more demanding; Bonato et al., 2012). Therefore, as the patients showed only very subtle symptoms even in these sensitive computer tests, a very plausible possibility is that adding further traditional methods to assess visual neglect would not change the present results. Nevertheless, further studies with larger batteries of tests are needed as they might bring additional information of the sensitivity of the present computer tests in revealing very subtle visuospatial symptoms.

A characteristic of the present study is the fact that we compared targets presented in the peripersonal space (dual-tasks, paper-and-pencil cancellation task) with targets presented in the extrapersonal space (large-screen cancellation task). It is possible that RH patients’ spatial bias was limited only to peripersonal but not to extrapersonal space, and this, rather than attentional demands, would explain the dissociation we found. However, in our previous study (Villarreal et al., 2020), the same RH patients showed subtle neglect in the extrapersonal space, as assessed with large-screen dual-tasks, and both studies demonstrated that paper-and-pencil cancellation tasks were not able to reveal spatial bias in RH patients. Hence, in our present and previous studies, RH patients did not show spatial bias in peripersonal or extrapersonal space in the cancellation tasks but did in both sectors of space in the dual-tasks. The present findings are supported by the single case analyses presented in Supplementary Table A. Statistically reliably contralesional (as compared to ipsilesional) omissions were evident in eight of the patients with the dual-task method but in none of the patients with the cancellation tasks. Most patients showing deficits were initially not diagnosed with neglect by using traditional methods, indicating that their symptoms were subtle already in the early phase of recovery.

To conclude, we compared the sensitivity of two different computer-based methods (cancellation large-screen vs. dual-tasking) and of one paper-and-pencil cancellation task, in detecting subtle visuospatial attention deficits in right hemisphere stroke. The dual-task approach outperformed the cancellation task approach when using paper and pencil but also on a large screen. Therefore, attentionally more demanding methods than conventional approaches are useful in revealing mild forms of visuospatial inattention. It is important to detect even these minor deficits since they may cause important real-life problems (see, e.g., Deouell et al., 2005; Bonato et al., 2012; Sotokawa et al., 2015).

## Data Availability Statement

The datasets presented in this article are not readily available because of the privacy and/or ethical restrictions. Individual requests for sharing the data will be considered after additional approval for sharing by the local ethics committee. Requests to access the datasets should be directed to SV, sanna.villarreal@helsinki.fi.

## Ethics Statement

The studies involving human participants were reviewed and approved by the Ethics Committee of Helsinki University Hospital. The patients/participants provided their written informed consent to participate in this study.

## Author Contributions

SV, ML, RS, HJ, and MH planned and initiated the study and planned the twinkle task. ML and RS planned and constructed the active space application, and responsible for the technical properties of the large-screen version. SV and OV constructed the paper-and-pencil version of the twinkle task and recruited participants and collected the data. MB planned and constructed the visual and auditory dual-tasks and related study-design. SV and MB planned the statistical approach. SV conducted the statistical analyses and drafted and finished the manuscript. All authors contributed to the critical revision of the manuscript and approved the final version to be published.

## Conflict of Interest

The authors declare that the research was conducted in the absence of any commercial or financial relationships that could be construed as a potential conflict of interest.

## Publisher’s Note

All claims expressed in this article are solely those of the authors and do not necessarily represent those of their affiliated organizations, or those of the publisher, the editors and the reviewers. Any product that may be evaluated in this article, or claim that may be made by its manufacturer, is not guaranteed or endorsed by the publisher.

## Footnotes

1
http://www.pstnet.com/
